# Editors' reply

**DOI:** 10.4103/0019-5413.40268

**Published:** 2008

**Authors:** Anil Kumar Jain

**Affiliations:** Editor, Indian Journal of Orthopaedics, Professor of Orthopaedics, University College of Medical Sciences and GTB Hospital, Delhi, India. E-mail: dranilkjain@gmail.com

Sir,

Thank you very much indeed for your kind words and your interest in the Indian Journal of Orthopaedics. The objective of organization of the symposium is to give a few state-of-the-art reviews based on the articles published in the literature and include a few original articles covering the ongoing research on the subject. The editorial tries to balance it keeping an eye on the problems of a particular geographical region.

There is unanimity in the literature about the role of conservative treatment for spinal column fracture and Spinal cord injury (SCI) patients and it is well documented in the textbooks. Including articles on rehabilitation program for SCI would have been a repetition of the information. The editorial not only stresses on the prevention of spinal trauma but also on the current scenario of the care of spinal trauma patients across the country. The problem of spinal trauma and SCI including initial evaluation and care of the patient, travel time to nearest hospital, infrastructure and quality of treatment at district hospital is covered. It also stresses on better organization of spinal trauma care services, achievable standard of spinal trauma services, and also on lack of treatment guidelines for neglected spinal trauma cases. I feel a journal has to be balanced in maintaining the minimum service quality while also continuing to educate the orthopedic community about future research in the field. The clinician needs to know which are the fractures best treated by the conservative approach and which would do better with surgical intervention, if intervention is indicated than what type of intervention. All dilemmas have been spelled out in the editorial and some of them are covered in original articles.

We have a ‘letter to editor’ column in the Indian Journal of Orthopaedics and would be keen to have more interaction between readers and authors. However, your points are valid and will be given a serious consideration for future issues of the Indian Journal of Orthopaedics.

